# The Great East Japan Earthquake: Experiences and Suggestions for Survivors with Diabetes (perspective)

**DOI:** 10.1371/4facf9d99b997

**Published:** 2012-05-15

**Authors:** Miyako Kishimoto, Mitsuhiko Noda

**Affiliations:** Chief, Department of Diabetes and Metabolic Medicine, Center Hospital, and Diabetes and Metabolism Information Center, Diabetes Research Center, National Center for Global Health and Medicine, Tokyo, Japan; Department of Diabetes and Metabolic Medicine, Center Hospital, and Diabetes and Metabolism Information Center, Diabetes Research Center, National Center for Global Health and Medicine, Tokyo, Japan

## Abstract

The Great East Japan Earthquake and the subsequent tsunami that occurred in the afternoon of March 11, 2011, destroyed large parts of Japan’s Tohoku district. Owing to the unfavorable living environment, many diabetic patients in the refuges lost control of their blood glucose levels, and in addition, the high-calorie food provided led to severe postprandial hyperglycemia. We recommend that diabetic patients keep personal stocks of medical supplies and the medication that they require daily, as well as records of their medication. We also recommend the creation of basic guidelines to facilitate the practical prescription of medication for diabetic patients under various conditions that may arise in the aftermath of a natural disaster.

## Introduction

In the afternoon of March 11, 2011 in Tokyo, we experienced a sudden, strong earth tremor. The floor kept rolling, and people were unable to remain standing. This earthquake reminded us of the Great Hanshin-Awaji (Kobe) earthquake in 1995 (M6.9) that killed more than 6400 people and left us deeply scarred. Soon after the quake, what we saw on television broadcasts was a manifestation of hell on earth: many houses and cars were swept away by the giant tsunami and nothing but devastated farms and residential areas were left behind in large parts of the Tohoku district. To make matters worse, the Fukushima nuclear power plant experienced a leak^1^ . This massive earthquake with a magnitude of 9.0 was named the Great East Japan Earthquake^2^. The disaster left tens of thousands homeless, and many people had to evacuate to refuges. In the end of April 2012, more than a year after the earthquake, the death toll is over 15,800, and more than 3,000 people are still missing^3^ .

## Observations

Our experiences in the affected area

The National Center for Global Health and Medicine (NCGM) of Japan immediately sent the Disaster Medical Assistance Team (DMAT), a group of professional medical personnel trained to provide rapid-response medical care, to Sendai in Miyagi prefecture of the Tohoku district, one of the affected areas. DMAT had completed its activities by March 16; thereafter, the NCGM’s medical support teams, composed of four to six members including doctors, nurses, pharmacists, clinical psychologists, and clerical staff were sent every two days. By the end of June 2011, more than 250 NCGM personnel from 51 teams had carried out their tasks in the affected area, in cooperation with local public health nurses. Unlike the Hanshin-Awaji quake, in which most of the casualties were due to the collapse of structures and subsequent fires, the tsunami itself caused most of the casualties. Therefore, the medical support teams were expected to provide care to patients with chronic diseases such as diabetes, hypertension, respiratory diseases, and mental diseases, rather than patients with acute diseases, for which the DMAT had provided treatment. The medical team made a round of visits to the refuges of Higashimatsushima City in Miyagi prefecture and also conducted a health survey for people who resided at home. Initially, we visited 14 refuges every 3 days and examined and treated more than 1,110 patients by the end of April 2011.

After a disaster, conditions such as stress, lack of food or water, extremes of heat or cold, and infection can contribute to rapid worsening of a chronic illness that was under control before the event^4^ ^5^. For many patients with diabetes, their blood glucose control was lost, and their general conditions deteriorated with the unfavorable living environments of these patients. Many patients had lost their medication records, and the medical team had no idea what treatments they had received before the disaster occurred. Therefore, the team carried a catalog of oral antidiabetic drugs with their photos and an insulin catalog with colorful photos, manufactured by the three major insulin companies; these catalogs helped patients to recognize and identify their previous medications, to some extent. Even when we could identify the former medication, we sometimes had to change the medications to maintain the patient’s blood glucose at relatively high levels and to avoid hypoglycemia, rather than maintaining optimal blood glucose levels with strict control, because grief due to the loss of family and property reduced appetite.

## Realistic Diets

Diet is a fundamental element of diabetes management. Living in refuges, however, made it very difficult for patients with diabetes to eat properly, because some refuges could not even provide basic healthy meals, let alone diets appropriate for patients with diabetes. The Japan Dietetic Association reported that even by the end of April 2011, almost a month and a half after the disaster, most of the refuges in Ishinomaki City and Onagawa-cho in Miyagi prefecture could provide only two meals per day: breakfast with bread and a cooked supper. In early April, 90% of the refuges in Kesennuma City provided three meals per day, but 40% of them could not provide meat and eggs, whereas 30% of them could not provide vegetables. In the cases of the Great Hanshin-Awaji Earthquake in 1995 and the Niigata Chuetsu Earthquake in 2004, people could receive packed lunches from the surrounding cities at a relatively early stage. However, in the case of the earthquake in Tohoku, the dietary circumstances did not rapidly improve for several possible reasons: the extremely vast size of the affected area, difficulty in transporting relief supplies due to the destruction of roads, railways, and airports, and relatively old age of the people living in the refuges (i.e. few people in the refuges were healthy enough to prepare meals). Some refuges had plenty of sweet buns, snacks, and rice balls that were high in carbohydrates; however, fresh vegetables and fruits from all over Japan remained packed in cardboard boxes and rotted because no one could cook and serve them. Unfortunately, precious food was not handled properly. Although the supplied meals were not always healthy for patients with diabetes and were often high in calories, the patients had no choice. Some patients showed severe postprandial hyperglycemia after consuming the meals supplied in the refuges. Common comments included, “Nobody knows when the next aftershocks will come. I may not be able to get a meal then. I want to eat while I can.” “I think I’ve been eating too much, but I can’t leave this because it is wasteful.” or “I’m afraid other people will see my leftovers and may think that I’m ungrateful.” One piece of advice we were able to give these patients was to share their meals with their families.

Dehydration increases the risk of DVT and aggravates diabetic control. Despite our suggestions regarding the consumption of sufficient amounts of liquid to avoid dehydration, elderly people tended to limit their liquid intake to reduce the number of times that they needed to use the lavatory, which was often far away, cold, and not sanitized.

## Mental Health Care

As reinforced by the World Trade Center disaster on September 11, 2001^6^, mental health care is one of the most important issues in disaster-stricken areas^6^ ^7^ ^8^. The acute memory of the disaster and associated fears are deeply engraved in the mind, causing mental trauma. In addition to emotional responses such as grief, loss, and anger, a person may be beset by a sense of heavy obligation for being the one who survived (survivor’s guilt). Most survivors, living in refuges experience social and lifestyle stress with regard to privacy, food, lavatory use, duty assignments, and care for others who need help. Therefore, mental support is critical to patients with any disease or irrespective of the existence of a disease, and acute mental care, such as counseling by a specialist, is sometimes necessary. Especially for diabetic patients, mental stress may aggravate diabetic control^9^ and special care is needed. Patients should be advised not to endure their worries or anxieties alone but to attempt to find someone with whom they can consult.

## Conclusions and Recommendations

As diabetologists, and based on our experiences of the Great East Japan Earthquake, we offer the following suggestions to cope with a disaster situation; (1) Maintaining daily stocks of necessary supplies and being mentally prepared for emergencies is crucial. Diabetic patients should keep stocks of their medication with them and record their medication and dosage regimens in a notebook, card, or media device. In addition, not only medical materials, optimal glycemic control in pre-disaster is important. (2) There are virtually no guidelines regarding the management of chronic medical conditions after natural disasters^10^. Basic guidelines need to be created to allow practical prescription of medication for diabetic patients under various situations in the event of a natural disaster. Self-monitoring of blood glucose may be helpful in determining the prescription. (3) Diet management after a disaster is very difficult; therefore, under the assumption of various cases, educating patients beforehand to enable them to make proper decisions based on their situation is important. (4) For diabetes management, a multidisciplinary medical team is indispensable. Our medical support team for the disaster in the Tohoku district kept close contact with each other, shared information, and worked with a single purpose. In the follow-up to this disaster, the provision of not only good immediate care to diabetic patients, but also long-term, continuous, and comprehensive care for these patients, including mental health support, will be necessary.

## Competing Interests

The authors have declared that no competing interests exist.
